# Physiotherapy students’ experiential learning through curriculum-integrated high-complexity simulation: a qualitative study

**DOI:** 10.1186/s41077-026-00474-3

**Published:** 2026-08-03

**Authors:** Mirari Ochandorena-Acha, Anna Escribà-Salvans, Ramon Jolis-Prat, Laia Briones-Buixassa, Cristina Vaqué-Crusellas, Sandra Rierola-Fochs, Vinicius Rosa Oliveira, Marc Terradas-Monllor

**Affiliations:** 1 Research Group on Methodology, Methods, Models and Outcomes of Health and Social Sciences (M3O), Faculty of Health Sciences and Welfare, Centre for Health and Social Care Research (CESS), University of Vic-Central University of Catalonia (UVic-UCC), Sagrada Familia 7, Vic, 08500 Spain; 2 Institute for Research and Innovation in Life Sciences and Health in Central Catalonia (IRIS-CC), Vic, Spain; 3 Technology, Inclusion and Health Research Group (TIS), University of Vic-Central University of Catalonia (UVic-UCC), Vic, 08500 Spain

**Keywords:** Simulation-based education, Physical therapy, Experiential learning theory, Education, Learning, Simulated patient, Higher education

## Abstract

**Background:**

Developing both technical and non-technical competencies is a core goal in physiotherapy education. While traditional clinical placements offer essential hands-on experience, they may not fully prepare students for the complexity and unpredictability of real-world clinical practice. Simulation-based education (SBE) can help bridge this gap by providing a safe, realistic, and structured learning environment.

**Objective:**

To explore undergraduate physiotherapy (PT) students’ experiences of curriculum-integrated high-complexity SBE, through the lens of experiential learning theory.

**Design:**

Qualitative descriptive study.

**Setting:**

Faculty of Health Sciences and Wellbeing, University of Vic – Central University of Catalonia, Spain.

**Participants:**

Twenty-eight undergraduate PT students from the second, third, and fourth academic years (fourth, sixth, and eighth semesters, respectively) participated in six focus groups conducted between June and October 2024. Participants were purposively sampled to ensure diversity in academic year and simulation roles (PT or observer).

**Exposure:**

High-complexity simulation sessions embedded in the PT curriculum, involving trained simulated patients and structured facilitation.

**Main outcomes and measures:**

Students’ experiences of learning processes during SBE. Data were analysed using reflexive thematic analysis and later interpreted in relation to experiential learning theory. ATLAS.ti software was used to support data management. Transcripts were returned to participants for member checking.

**Results:**

Two overarching themes emerged: (1) *Constructing non-technical skills through action*,* observation*,* and reflection*, and (2) *Experiencing safety and immersion*. Students valued both performing and observing roles, reporting that observation fostered reflection, vicarious learning, and reduced performance anxiety. Structured debriefings, progressive exposure, and peer feedback reinforced learning, while facilitators’ guidance and psychological safety promoted confidence. The use of simulated patients enhanced emotional immersion and authenticity, and students highlighted their potential role in feedback.

**Conclusions:**

High-complexity SBE was perceived as a meaningful and situated learning experience that supported reflection, knowledge integration, and application, contributing to the development of non-technical skills and confidence.

**Trial registration:**

NCT06852482 (clinicaltrials.gov).

**Supplementary Information:**

The online version contains supplementary material available at https://doi.org/10.1186/s41077-026-00474-3.

## Introduction

In physiotherapy (PT) education, the development of both technical and non-technical skills is the primary goal of the curriculum [1, 2]. Traditionally, clinical placements have been regarded as the most effective setting for fostering these competencies [3, 4]. Yet, students often report that the classroom-based teaching preceding these placements does not sufficiently prepare them for the realities of clinical practice [5, 6]. Over the last decade, the use of simulation-based education (SBE) in PT curricula has expanded, as it is increasingly recognised as an effective and safe approach to recreate authentic, context-specific scenarios in a controlled environment [7–9]. This enables learners to engage in patient care without exposing real patients to risk, while bridging the gap between theoretical learning and real clinical practice [7].

Simulation experiences range from peer role-play to interactions with trained simulated patients (SPs) [8, 10]. SPs are widely recognised as a valuable and effective resource for teaching and assessing communication and physical examination skills in healthcare students, as they can create realistic, interactive, and emotionally engaging learning encounters [8, 10]. These highly authentic learning experiences, while challenging, may offer authentic opportunities to develop professional competencies [11, 12]. However, although existing evidence highlights gains in skill acquisition and student satisfaction [13], less is known about the processes through which SBE fosters professional competency development in PT students. In particular, the experiential and reflective dimensions of learning during SP-based SBE remains underexplored, especially among undergraduate PT students.

Learning in clinical education is not solely the result of performing skills; it also requires opportunities for reflection, conceptualisation, and application in context [14]. Kolb’s experiential learning theory provides a valuable framework to understand these processes, describing learning as a cyclical progression through four interrelated stages: first, *concrete experience* (actively engaging in a specific task or activity), then, *reflective observation* (reviewing and critically considering the experience from different perspectives), next, *abstract conceptualisation* (integrating observations into existing knowledge and developing new concepts or principles), and finally, *active experimentation* (applying these concepts to new situations or modifying one’s approach). This framework has been widely applied in healthcare education [14, 15], yet the specific contribution of these elements to PT students’ learning through SBE has received little attention. Furthermore, although simulation has been increasingly examined in physiotherapy education, much of the literature has focused on single simulation events, individual courses, or discrete educational outcomes [16, 17]. Less is known about how students experience high-complexity SBE when it is embedded progressively across the undergraduate curriculum. Few qualitative studies have examined how PT students experience high-complexity SBE [16, 18]. Gaining insight into how students navigate these experiences across different academic years may inform the design of simulation activities that are both pedagogically and professionally relevant. Therefore, this qualitative study aimed to explore how undergraduate PT students experienced curriculum-integrated high-complexity SBE across different academic years, through the lens of experiential learning theory.

## Methods

### Study design

This qualitative study adopted an interpretative approach informed by Kolb’s experiential learning theory [14, 19]. The framework provided a theoretical lens for exploring how students made sense of their simulation experiences and supported the subsequent interpretation of the findings. Data were analysed using reflexive thematic analysis, following Braun and Clarke’s approach [20]. The choice of a qualitative design was particularly appropriate for capturing the complexity of students’ subjective experiences, an approach that has been highlighted as essential for advancing PT education research [21].

To ensure the study’s quality and rigour, the Standards for Reporting Qualitative Research (SRQR) were followed, applying established criteria for trustworthiness in qualitative research, including credibility, transferability, dependability, and confirmability. The reporting of methods and findings was structured in line with these standards to enhance transparency and reproducibility [22].

The study was conducted in accordance with the ethical principles of the World Medical Association Declaration of Helsinki [23]. Ethical approval was granted by the Research Ethics Committee of the University of Vic – Central University of Catalonia (UVic-UCC) (292/2023), and the study was registered in the Clinical Trials Registry (NCT06852482). All participants were informed about the study aims, confidentiality, and data handling procedures. Written informed consent was obtained before participation, including consent for audio recording, transcription, and the anonymous use of quotations in publications.

### Participants and recruitment

The study was conducted at the Faculty of Health Sciences and Wellbeing, University of Vic – Central University of Catalonia, where undergraduate PT education follows a four-year (eight-semester) curriculum integrating theoretical instruction, laboratory-based practice, and clinical placements.

A purposive sampling strategy was used [24] to ensure demographic variability and adequate information power [25]. Eligible participants were 18 years or older, officially enrolled in one of the courses implementing SBE (second, third, or fourth academic years), and had participated in the corresponding simulation sessions during the 2023–2024 academic year. Participant selection considered age, gender, academic year, and previous simulation role experience (PT and observer) to enhance sample diversity and capture a broad range of perspectives. Focus groups were organised separately by academic year to facilitate discussion among students with comparable curricular exposure, while purposive sampling within each group sought variation in gender, age, and prior simulation role experience.

Recruitment took place after the completion of all SBE sessions included in the academic program. A member of the research team carried out the participant selection process. Initial contact was made via an email sent by the first author, who was also the responsible for the courses in which the SBE were conducted during that academic year. The message was previously reviewed by the research team to minimise potential perceptions of coercion or imbalances in power dynamics between researchers and students.

As a moderate level of non-attendance was anticipated, a slightly higher number of students were invited to ensure the effective number of participants per focus group was reached (approximately eight per academic year). Students who expressed interest received detailed written information about the study, including ethical considerations such as confidentiality and the right to withdraw at any time. Students who declined participation, did not provide informed consent, failed to respond to follow-up communication, or did not attend the focus group were excluded.

After the focus groups (FGs), participants received a small gift voucher as a token of appreciation for their voluntary participation and time. This token was not mentioned during recruitment to avoid influencing participation.

### Simulation experience

SBE is embedded throughout the undergraduate PT program as a bridge between classroom learning and clinical practice, following the International Association for Clinical Simulation and Learning (INACSL) Standards of Best Practice for simulation design, facilitation, assessment, and debriefing [26–31]. Students engage in progressively complex SBE throughout the curriculum, ranging from peer role-play in early courses to high-complexity simulation scenarios with trained SPs during the second, third, and fourth years (corresponding to the fourth, sixth, and eighth semesters, respectively).

In the present study, the term high-complexity SBE refers to the simulation experiences involving trained SPs, complex clinical scenarios, integration of technical and non-technical competencies, and increased demands for communication, clinical reasoning, adaptability, and autonomous decision-making. These simulations, facilitated by trained lecturers (hereafter referred to as facilitators), included structured phases of prebriefing, scenario performance, and debriefing, and addressed learning objectives related to communication, clinical skills, and reasoning.

During the programme, students complete one mandatory high-complexity simulation in each academic year from the second to the fourth year. Therefore, by the time of participation in this study, students in the second, third, and fourth academic years had accumulated one, two, and three high-complexity simulation experiences, respectively. Role participation was documented across sessions to support rotation between active and observer roles. All students were expected to assume the PT role at least once during their cumulative simulation pathway, while observer participation in remaining sessions was designed as an active learning role supported by structured observation tasks and debriefing. Students could express role preferences, and facilitators encouraged role allocation that promoted equitable participation and prior role balance.

Scenario complexity and learner autonomy increased gradually across academic years. In the second year, the PT role was performed in pairs, and prebriefing sessions were longer and more structured to promote psychological safety and clarify objectives. In the third and fourth years, students performed individually, facing more complex and unpredictable cases, while debriefings were extended to promote deeper reflection and self-evaluation. Simulations were conducted in small groups of five students, with peers assuming the observer role and following the scenario via live audiovisual projection using standardised observation sheets. Scenario complexity also progressed across academic years, in alignment with the undergraduate PT curriculum, encompassing orthopedic, neurological, cardiovascular, pediatric, and community health cases in simulated outpatient and long-term care settings. To provide a clearer overview of how simulation is structured and progressively integrated across the curriculum, Table 1 summarises the key characteristics of high-complexity SBE across academic years.

**Table 1:** Integration and progression of high-complexity SBE across the undergraduate PT curriculum

Academic year	Semester	SBEs per academic year	No. of high-complexity SBE (cumulative*)	Simulation format	Student role	Facilitator support	Key learning focus	Progression characteristics
2nd year	4th	1	1	SP-based simulation	PT in pairs + observers	High (structured guidance)	Communication in complex patients; managing unexpected events; introductory clinical reasoning	Lower complexity, structured guidance. Emphasis on psychological safety and confidence building.
3rd year	6th	1	2	SP-based simulation	Individual PT + observers	Moderate (shared responsibility)	Integration of biopsychosocial factors; evidence-based reasoning; adaptability	Moderate complexity. Increasing autonomy, balanced facilitation, deeper reflection
4th year	8th	1	3	SP-based simulation	Individual PT + observers	Low (facilitator as guide)	Advanced clinical reasoning; interdisciplinary collaboration; intervention planning	Higher complexity. Self-directed reflection, preparation for clinical practice

*PT* Physiotherapist, *SBE* Simulation-based education, *SP* Simulated patients

*By the end of the second, third, and fourth academic years, students had completed one, two, and three cumulative high-complexity SBE experiences, respectively

### Data collection

Data were collected through face-to-face FGs which provided a space for students to reflect on their experiences with SBE, allowing for rich, in-depth discussion. FGs were developed between June and October 2024 at the UVic-UCC.

A semi-structured interview guide was developed based on the researchers’ expertise (see Appendix 1 in Supplementary material). The guide was piloted in an individual semi-structured interview with a fourth-year PT student who met the participant profile but was not included in the final sample. This pilot interview was used to assess question clarity, flow, and relevance, leading to minor refinements in wording, sequencing, and prompts. It included open-ended questions and was administered in either Spanish or Catalan, according to each group’s language preference.

FGs were independently conducted by three members of the research team: a psychologist (L.BB.), a nutritionist (C.VC.), and a physiotherapist (R.JP.). All were experienced university lecturers, and none were actively involved in teaching the participants at the time of data collection, ensuring a neutral and non-evaluative environment. All three interviewers were experienced in qualitative interviewing and received specific preparatory training before data collection. This consisted of a two-hour calibration session in which the research aims were reviewed, the interview guide was discussed question by question, and responses from the pilot interview were jointly examined to refine prompts, clarify wording, and promote consistency in facilitation style across FG. Furthermore, interviewers were provided with a brief facilitation manual containing an opening script, recommendations for session closure, and example probing questions to support deeper exploration of participants’ perspectives. Fluent in both Spanish and Catalan, they adopted a receptive and supportive style that created a relaxed atmosphere, ensured balanced participation, manage dominant voices, and encouraged students to elaborate freely. In cases where participants experienced distress, the interviewers responded with empathy. Each session followed a structured format comprising an introduction, guided discussion, and closing segment.

All FGs were audio-recorded using two devices to prevent data loss. Interviewers also took field notes to capture contextual and non-verbal information, which were integrated into the transcripts as annotations. The recordings were transcribed verbatim using NVivo Transcription software and subsequently anonymised during the review phase by removing any identifying information. Each participant and FG were assigned a numerical code for confidentiality.

Final transcripts were returned to participants for member checking; however, none provided additional feedback, comments or corrections. All data—transcripts, recordings, and consent forms—were securely stored using the University’s Microsoft Office 365 infrastructure, protected by multi-factor authentication, encrypted transfers, and routine backups. Access to the data is restricted to the main author, who is responsible for their secure management and confidentiality.

Data collection concluded when data saturation and sufficient information power were achieved, considering the study’s focused aim, the specificity of the participant group, and the quality and depth of the data [25, 32].

### Data analysis

Data were analysed using reflexive thematic analysis (RTA), following the approach described by Braun and Clarke [20]. This method involves developing, analysing, and interpreting patterns across qualitative datasets through systematic coding and theme generation. The analysis was conducted collaboratively by the first (M.OA.) and third (R.JP.) authors, both members of the project’s research team, following six phases of RTA consistently across all transcripts: (1) familiarising with the data; (2) generating initial codes; (3) constructing candidate themes; (4) reviewing and refining themes; (5) defining and naming themes; and (6) writing the results.

The analysis was theoretically informed by experiential learning theory, which conceptualises learning as an active and contextual process of meaning-making through concrete experience, reflection, conceptualisation, and experimentation [14]. However, no fixed a priori codebook was developed. Instead, the framework functioned as a set of sensitising concepts that informed early analytic attention to how students described learning processes, while remaining open to unexpected meanings and patterns within the data. Initial coding was therefore flexible and iterative.

Themes were primarily developed inductively through close engagement with participants’ accounts and constant movement between coded extracts and the full dataset. Once themes had been generated and refined, they were interpreted in dialogue with experiential learning theory to explore how students’ narratives related to processes such as action, observation, reflection, confidence-building, and progressive participation. Thus, the final themes were theoretically enriched during interpretation through the lens of Kolb’s experiential learning theory.

Data analysis was conducted using ATLAS.ti software (version 24.1.1). Data were analysed in their original language (Spanish or Catalan), and selected illustrative quotes were subsequently translated into English for reporting.

### Reflexivity

The research team acknowledged the potential influence of their professional backgrounds and experiences on data collection and interpretation. All authors have expertise in PT education and research, along with experience in high-complexity SBE. The first author (M.OA.) has advanced expertise in qualitative research methods. The diverse disciplinary backgrounds of the authors (PT, psychology, and nutrition) contributed to a broader perspective in interpreting the results. To minimise bias, the authors reflected critically on their positionality throughout the analysis. Regular discussions were held to challenge assumptions and ensure that interpretations remained grounded in the students’ accounts.

### Role of the funding source

The funder played no role in the design, conduct, or reporting of this study.

## Results

A total of twenty-eight undergraduate PT students participated in the study across six FGs (ranged from 3 to 8 participants per FG), which lasted between 68 and 112 min. Participants were enrolled in the second (*n* = 4), third (*n* = 16), and fourth (*n* = 8) academic year of the undergraduate PT program. As high-complexity SBE was progressively embedded across the curriculum, students in the second, third, and fourth academic years had completed one, two, and three cumulative high-complexity simulation experiences, respectively, by the time of data collection. All particiapnts had taken part in at least one simulation during the 2023–2024 academic year, either in the active PT role or as a structured observer, with cumulative role rotation across the programme ensuring that all students experienced the PT role at least once.

Table 2 presents participants’ characteristics according to the established eligibility criteria, showing both homogeneity and heterogeneity of the sample, as well as the demographic composition of each FG.

**Table 2:** Sociodemographic characteristics of participants and focus group composition

Variable	Total sample (*n* = 28)	FG1 (*n* = 8)	FG2 (*n* = 5)	FG3 (*n* = 3)	FG4 (*n* = 3)	FG5 (*n* = 4)	FG6 (*n* = 5)
Age, years, mean (SD)	21.58 (3.38)	21,0 (2,8)	21,4 (2,6)	21,1 (2,5)	23,6 (3,0)	19,5 (2,4)	23,4 (2,9)
Gender, n (% of students)							
Female Male No binary	13 (46.43) 15 (53.57) 0 (0)	4 (50) 4 (50) 0 (0)	4 (80) 1 (20) 0 (0)	1 (33) 2 (67) 0 (0)	2 (67) 1 (33) 0 (0)	1 (25) 3 (75) 0 (0)	1 (20) 4 (80) 0 (0)
Undergraduate physiotherapy course, *n* (% of students)							
Second academic year Third academic year Fourth academic year	4 (14.29) 16 (57.14) 8 (28.57)	0 (0) 8 (100) 0 (0)	0 (0) 5 (100) 0 (0)	0 (0) 3 (100) 0 (0)	0 (0) 0 (0) 3 (100)	4 (100) 0 (0) 0 (0)	0 (0) 0 (0) 5 (100)
Role assumed during the last simulation, n (% of students)							
Physiotherapist role Observer role	13 (46.43) 15 (53.57)	3 (37) 5 (63)	0 (0) 5 (100)	3 (100) 0 (0)	1 (33) 2 (67)	2 (50) 2 (50)	4 (80) 1 (20)

*FG* Focus group, *SD* Standard deviation

The analysis generated two overarching themes and several related subthemes that captured how PT students experienced learning through high-complexity SBE. Together, these themes reflect the importance of active participation, observation, psychological safety, realism, and progressive challenge in shaping students’ learning experiences. Each theme is presented with illustrative quotes to support interpretation, while highlighting nuances within and across FGs (Table 3). Participant codes are used to preserve anonymity.

**Table 3:** Illustrative participant quotes supporting themes and subthemes identified in the reflexive thematic analysis

Quote ID	Theme / subtheme	Quote	Participant code	Focus group code
Q1	Action, observation and reflection > Roles	I mean, because you feel like the protagonist in that moment [*when participant referred to being in the role of PT during simulation*], when you play the physiotherapist. When you are the observer, you also feel like the protagonist, but in a different way, because you have the ability to see and critically reason about what is being done well, what is being done poorly, and you can learn from it. You can have both positive and meaningful learning, and negative learning—by negative learning, I mean things you would not want to apply yourself.	P01, 4th year student	FG04
Q2	Action, observation and reflection > Roles	I think you learn more when you are the physiotherapist because you experience everything first-hand. Later, during the debriefing, you realise what you did wrong because you’ve lived it yourself. When you’ve gone through it personally, you understand it better than anyone else—and when you listen to the feedback, you can really integrate it and apply it the next time.	P01, 3rd year student	FG01
Q3	Action, observation and reflection > Roles	The simulation allowed me to become more aware, to gain confidence, and to reflect. [*When in the PT role*] Beyond self-confidence, it was also about being aware that someone was watching me, taking notes (…) which can affect how I perform. [*When in the observer role*] When I see other people working, I notice how they organise their sessions. I compare it with my own way of organising—if I do it in a similar way, if it works as well as theirs. This is more about organising the process. Therefore, whether acting as the physiotherapist or as an observer, you reflect on how you could structure the session. As an observer, I might think “I would start with this, then do that,” while the other person does things in a different order—but in the end, it leads to the same outcome. (…) I think there is a ‘before’ and an ‘after’ in our learning.	P03, 4th year student	FG04
Q4	Action, observation and reflection > Roles	I think you learn just as much when you observe, because while watching the physiotherapist you’re also thinking, “Oh, I would have done this.” But then you realise that if you had done that, the patient might have responded differently—and that changes the whole interaction. So, as you follow the session, you keep reflecting and adjusting in your mind.	P01, 4th year student	FG06
Q5	Action, observation and reflection > Debriefing for learning	For me, the most valuable part was not just the simulation itself, but the debriefing afterwards. We went through a timeline on the board, discussing what could have been done better, embracing both the good and the bad, strengthening what went well and improving what didn’t. It was the most extensive part of the session and where you could truly learn whether you had been in the physiotherapist role or observing.	P03, 3rd year student	FG01
Q6	Action, observation and reflection > Debriefing for learning	The feedback never felt like criticism—it was constructive, aimed at helping us improve. Both the facilitator and the observers [*refering to peers*] highlighted what we did well, as well as what could be better, which made it easier to speak openly, reflect, and participate.	P01, 3rd year student	FG03
Q7	Action, observation and reflection > Confidence and readiness	I think the main advantage is the self-confidence it gives you. When you do it and then see that it went well, it’s like a boost of security and empowerment—it feels great. It’s the closest thing we have to real practice apart from clinical placements. Many people finish their degree with the knowledge but without confidence, saying “I wouldn’t know how to do that.” Simulation gives you that trial run, and by trying it, you gain the confidence you’ll need in the future.	P03, 4th year student	FG06
Q8	Action, observation and reflection > Confidence and readiness	For me, the simulation helped me gain awareness and confidence. I think there’s a *before* and *after… A*t the beginning I was very nervous, but during the debriefing I realised that it had actually gone quite well.	P04, 4th year student	FG04
Q9	Action, observation and reflection > Unpredictable scenarios	[*While asking for positive aspects of simulations*] It was positive because it was very enriching. I came in prepared, but even when you’re prepared, everything can turn around completely [*laughs*]. Then you see whether you have the ability to turn things around yourself too [*referring to adapting to unexpected changes during the simulation*]. That was a positive aspect for me.	P02, 4th year student	FG04
Q10	Action, observation and reflection > Unpredictable scenarios	It was about learning, because this is what we’ll face in a couple of years — knowing how to stay balanced. Imagine if, in my case, no one had told me the patient was going to fall [*students were informed about the case they were going to have during the simulation*]; if that happened in real life, I would have been in shock, like ‘what just happened?!’ But now I know that anything can happen with a patient, and you have to stay alert and steady on your side.	P01, 2nd year student	FG05
Q11	Safety and immersion > Facilitator	One important thing is that they [*referring to the facilitator*] also explain the objectives they want us to address. Well, all these objectives—above all—give us a lot of peace of mind, and we knew we could ask for help with anything. It’s not as if they just leave you there without talking to you; they also interacted with us and gave us feedback. That’s important.	P02, 3rd year student	F01
Q12	Safety and immersion > Facilitator	*[Interviewer asks about the prior relationship with the facilitator]* I think it is important to know them beforehand—it helped me because it gave me confidence. It made it easier not to feel embarrassed about saying things or making mistakes. You can say something, but if you’re afraid of saying it wrong and you think the lecturer is a bit of a “jerk” (…), you just say, “okay, I’ll keep quiet.” But if they make you feel confident, then you want to speak and participate. She encouraged us to talk and take part— “don’t be afraid.” That’s very good; I really like that part.	P02, 3rd year student	F01
Q13	Safety and immersion > Facilitator	What really gave me calm was when the facilitator said, “What happens during the simulation stays here.” Knowing she wasn’t going to tell the other lecturers what we did well or wrong, and it made me feel more confident, because I could try things, make mistakes, and still feel safe to learn from them.	P03, 3rd year student	FG02
Q14	Safety and immersion > Progressive structure	As we did the simulation in pairs, it felt different and gave us more confidence—if I forgot something, my partner could correct me and help. When you’re alone, you feel you have less support, even if everything goes well. As these were our first simulation-based practices, the cases weren’t too difficult, but working in pairs was helpful for the experience.	P02, 2nd year student	FG05
Q15	Safety and immersion > Progressive structure	The first time, you don’t know what to expect. I think one very positive aspect is having to do it with a partner the first time. I don’t know if that was planned for that reason, but it really helps.	P03, 2nd year student	FG05
Q16	Safety and immersion > Emotional immersion	Once you’re in and have started, you even forget that the simulated patient is actually an actor, and that other people are watching you.	P03, 2nd year student	FG05
Q17	Safety and immersion > Simulated patient and setting	One of the best things was having the simulated patient played by an actress, someone we didn’t know, not a lecturer we had seen before or a classmate, like in role-playing. It made a big difference that it wasn’t a peer or a teacher. Also, doing the simulation in a separate room, not on video, and without people watching, without five classmates in the background, helped a lot. In class, we’ve done a lot of role-playing, but it’s always between ourselves, with everyone watching, and maybe you don’t take it as seriously.	P01, 3rd year student	FG01
Q18	Safety and immersion > Simulated patient and setting	Applying it in a real-type case, like with an actress or similar, is much better than doing it between classmates. It’s not the same, because they’re your peers. You might say something, but in reality, a real patient wouldn’t play out that way. With real “patients”, you have to adapt to them, think, “I can go this way, but not that way”, you need to improvise but thinking at the same time. So, practicing with a real patient is better.	P02, 3rd year student	FG03
Q19	Safety and immersion > Materials and realism	We already knew everything about the patient before they arrived—his whole history, even the diagnosis. In real life, you only know what’s in the chart, and then you have to ask and discover. It would feel more real that way.	P06, 3rd year student	FG01
Q20	Enhancing feedback > Incorporating SP in debriefing	One great thing would be for the actress to also take part in the debriefing, giving her opinion, like saying, ‘When you told me this, it made me want to go for a walk,’ or ‘You really motivated me when you said that, even though I was acting, you genuinely motivated me.’ Both the positive and the negative feedback from the patient’s perspective would be very valuable.	P04, 3rd year student	FG01
Q21	Integrating more simulations > Students’ demand	I think we should do more simulations, you forget what you did in first year in the PT role and might not do another until fourth year, or never [*referring to repeat the PT role*]. It would be great to have more simulations throughout the years, with different formats: one with video, another with a simulated patient, or even two physiotherapists working together while someone distracts you. This way, you could really see your progress over time.	P07, 3rd year student	FG01

Quotes are reproduced verbatim, with minor edits for readability (e.g., removal of filler words) while preserving original meaning. Parentheses (…) denote omitted text that was irrelevant to the research question. Square brackets […] contain clarifying information added by the authors (e.g., when participants referred to earlier parts of the discussion). Each quote is labelled with the Participant code (P) and the Focus Group code (FG)

### Constructing non-technical skills through action, observation, and reflection

This theme highlights how students described learning through active participation, peer observation, feedback, and reflection. These interconnected processes appeared to support growing clinical confidence and readiness for practice.

Findings within this theme suggest an evolving learning process in which participation, observation, and reflection contributed to increasing confidence and preparedness. First, students described how learning emerged through performing, observing, and reflecting on simulated encounters. This process laid the foundation for a growing sense of self-efficacy and professional assurance. As they gained confidence, students also recognised their improved ability to manage unpredictable or emotionally charged clinical situations. Finally, the perceived value of these experiences fostered a strong desire for more frequent and varied simulation opportunities throughout their education.

- Learning through doing, observing, and reflecting. Students reported that their understanding of patient interaction evolved not only by performing in simulations (*experiential learning*), but also by observing their peers reflecting collaboratively on the experience (*reflective observation*). They emphasised that both roles – PT and observer – offered distinct yet complementary learning opportunities (see Q1 in Table 3).
  While acting as the PT, students gained first-hand experience, although sometimes testing their skills under pressure, as they reported feeling anxious or nervous in this role (see Q2 in Table 3).
  As observers, they experienced less performance anxiety, which allowed them to notice subtle relational dynamics, communication patterns, and clinical reasoning strategies that might otherwise go unnoticed. In fact, observation was not perceived as passive, but an active learning process that encouraged critical thinking and self-awareness: comparing how others organised their session helped them refine their own reasoning and professional approach (see Q3 and Q4 in Table 3).
  This interplay between action and observation was further strengthened through structured debriefing (*facilitated reflection*), which created space for reflection, discussion, and integration of lessons learned. Students highlighted this stage as the most valuable part of the session, where they analysed what worked and what could be improved, reinforced good practices, and learned constructively from mistakes (see Q5 in Table 3).
  The presence of psychological safety, fostered by the facilitator during the prebriefing and reinforced through constructive feedback from both peers and the facilitator, encouraged open discussion and helped students feel supported to express doubts and acknowledge errors without fear of judgment (see Q6 in Table 3).
- Building clinical confidence. Students consistently reported that SBE strengthened their self-confidence and sense of preparedness. Working through realistic scenarios in a non-evaluative setting, receiving immediate constructive feedback, and reflecting during debriefing led to a shift from caution to willingness to act. These gains were described as cumulative—built through repetition, progressive complexity, and the chance to “try things out” before clinical placements (see Q7 in Table 3).
  Beyond technical performance, confidence often emerged from overcoming initial nervousness and realising, through reflection, that they were capable of managing complex situations successfully (see Q8 in Table 3).
- Improved readiness to manage unpredictable scenarios. Simulation exposed students to realistic, often unpredictable situations that required them to adapt in real time, manage stress, and make quick yet thoughtful decisions. Several students described being challenged to respond to the uncertainty and emotional demands of clinical encounters—an experience that ultimately proved highly valuable for their learning (see Q9 in Table 3). Similarly, other reflections illustrated how simulation fostered not only procedural competence but also mental preparedness to remain calm and responsive under pressure (see Q10 in Table 3).

### Experiencing safety and immersion

This theme reflects how PT students experienced SBE as a learning environment that combined psychological safety with immersive realism. These interconnected dimensions appeared to facilitate emotional engagement, authentic participation, and preparation for future clinical practice.

- Creating a safe and supported environment. Participants consistently described the experienced simulations as a safe, realistic, and progressively structured (*safe learning environment*), which encouraged active participation and learning through doing. Students valued facilitators who clearly explained the objectives of each simulation and maintained an interactive supportive presence throughout the sessions. This guidance gave them a sense of calm and direction, as they knew what was expected and felt comfortable asking for help (see Q11 in Table 3). They appreciated that mistakes were welcomed as learning opportunities, supported by facilitators who were emotionally reassuring, non-judgmental, and trustworthy, creating the confidence needed to fully engage in the scenarios.
  The presence of a familiar facilitator, who had already accompanied them in previous courses, further contributed to this sense of safety and trust (see Q12 in Table 3). The facilitator’s explicit reassurance of confidentiality, usually delivered during the prebriefing, was also deeply valued. Students appreciated knowing that what happened in the simulation would not be shared with other faculty members or used for assessment purposes (see Q13 in Table 3).
  In addition, the progressive structure of the sessions—starting the PT role in pairs during the second course—allowed students to gradually build their confidence and familiarity with the format. This incremental exposure aligned with experiential learning principles, helping students feel prepared to act independently in more complex scenarios (see Q14 and Q15 in Table 3).
- Emotional immersion and realism. One of the most striking features of the simulation experience was the level of emotional immersion elicited by the SP. Several students described being drawn into the scenarios to the point of “forgetting it was a simulation” (see Q16 in Table 3). Students also consistently emphasized the stark difference between these actor-based simulations and peer-based role-playing (see Q17 and Q18 in Table 3). While peer simulations were useful for practising basic skills, their realism was sometimes questionable, and they were often perceived as artificial, less reflexive, and emotionally flat. In contrast, simulations with trained actors were seen as authentic, unpredictable, and emotionally rich. The authenticity of the SPs’ behaviours and reactions made the encounters feel real, prompting genuine emotional and cognitive engagement from students.
  The realism of the physical environment was described as a key factor in promoting immersion. Students emphasised that the simulation setting and even the layout of the room contributed to feeling as if they were in a real clinical environment. The separation from regular classrooms and the use of professional equipment and language helped them “enter the role” and envision themselves as future practitioners. However, some students felt that the extensive written case information provided beforehand slightly reduced the realism, as it limited the sense of discovery and spontaneity that characterises real patient encounters (see Q19 in Table 3).
- Potential future role of actors in feedback and debriefing. Several students expressed the desire for actors to play a more active role in the debriefing process. Beyond just acting, they saw value in hearing the actor’s perspective—how the “patient” felt during the encounter, whether they felt heard, respected, or understood (see Q20 in Table 3). This kind of dialogical feedback was viewed as a potentially rich source of learning, particularly for developing empathy and communication skills.
- Desire for increased exposure to SBE in future training. The perceived value of simulation led many students to express a strong desire for more frequent and varied simulation experiences throughout their training (see Q21 in Table 3). Students viewed SBE not merely as preparation for clinical placements, but as a transformative learning space that nurtured both competence and confidence. Their collective demand for more simulation-based experiences underscores the importance of embedding SBE longitudinally within PT curricula to consolidate academic identity and readiness.

## Discussion

This qualitative study explored how undergraduate PT students experienced high-complexity SBE embedded progressively across the curriculum, focusing on how they made sense of learning through action, observation, and reflection. The findings suggest that students perceived SBE not merely as an isolated instructional technique, but as a meaningful and cumulative learning experience that that supported the integration of theory and practice across different stages of their training. By examining students’ perspectives across academic years, this study contributes to the literature by moving beyond single-event or single-course evaluations of simulation and offering insight into how curriculum-integrated SBE may support experiential learning, confidence, and professional development in undergraduate PT education.

Aligned with Kolb’s experiential learning theory [14], our findings illustrate how students moved through the full experiential cycle: engaging in concrete experiences (as performers or observers), reflecting critically during structured debriefings, connecting those experiences to theoretical knowledge, and expressing readiness to apply their learning in clinical contexts. The debriefing process played a pivotal role in fostering this reflection, allowing students to analyse their actions, exchange perspectives with peers, and integrate facilitator feedback into their professional reasoning. Overall, simulation was perceived as a developmental process in which emotional engagement, contextual realism, and peer interaction converged to shape a dynamic and situated learning space.

A central component of this educational methodology was the safe learning environment created by the facilitators and the structured, progressively complex format of the sessions. This aligns with evidence from a systematic review by Garden et al. [33], which identified debriefing as the most influential phase in SBE for developing non-technical competencies such as communication, teamwork, and self-awareness. Students valued facilitators as both pedagogical guides and emotional anchors who established trust, particularly through prebriefing strategies such as confidentiality and clear expectations, and whose presence they often already knew from previous courses. Similarly to previous research [34, 35], this familiarity contributed to psychological safety, reducing anxiety and enabling students to engage more authentically.

Consistent with the Planning, Implementation, Evaluation, Revision (PIER) framework [36], a model that conceptualises simulation as a cyclical and iterative process of design, facilitation, and reflection, our findings underscore the adaptive nature of facilitation and the importance of both theoretical and psychological preparation to foster meaningful engagement in SBE [26]. The PIER framework conceptualises facilitation as a dynamic process that evolves across simulation phases – from planning and briefing to debriefing and revision [36]. It also emphasises the need for deliberate student preparation, which our findings reinforce: students reported that understanding learning objectives in advance, feeling supported during prebriefing, and being reassured about confidentiality enabled them to participate more authentically [36]. In our program, the progressive structure of simulations—from paired participation in early years to individual practice in later stages—mirrored the PIER emphasis on scaffolding learning experiences through iterative cycles of planning, implementation, and reflection. Furthermore, constructive feedback and opportunities for deliberate practice aligned with the *evaluation* and *revision* phases of the framework, allowing students to critically appraise their performance, integrate feedback, and plan how to apply learning in future clinical situations.

The use of SPs further enriched SBE environment by fostering realistic, emotionally engaging interactions that mirrored clinical complexity. In this line, previous evidence in health professions education have shown that SP-based simulations promote to strengthen communication, empathy, clinical reasoning, and patient-centred interaction skills [37, 38]. Our findings suggest that SP involvement in PT training similarly promotes the development of relational and emotional competencies within a psychologically safe learning environment. In this line, several students expressed interest in receiving feedback directly from SPs, particularly regarding the patient’s perceived emotions and sense of being understood. Although this was not included in the current simulation format, these results point to the potential value of integrating SP feedback in future iterations to enhance reflection, satisfaction, and overall learning quality.

Consistent with prior studies, students clearly distinguished between learning through peer role-play and simulations involving trained actors [10, 39]. Peer role-play was described as useful for practising basic techniques but were often perceived as artificial and emotionally limited. In contrast, high-complexity simulations offered authentic, unpredictable, and emotionally rich encounters that challenged students to adapt and regulate their responses in real time. These findings echo evidence from Pritchard et al. [10], who reported that SPs provide more immersive and emotionally salient experiences than peer role-play. While empirical comparisons between high-complexity simulations and other learning modalities remain scarce, our findings reinforce their complementary pedagogical value, particularly in fostering communication, reflective practice, and professional identity formation.

Importantly, students highlighted the dual value of participating both as performers (PT role) and as observers. Consistent with literature [13, 39–41], the observer role was not perceived as passive but as an active and powerful pedagogical strategy that reduced performance anxiety, fostered critical reflection, and promoted vicarious learning. Watching peers interact with SPs enabled students to recognise subtle interpersonal cues, evaluate strategies critically, and mentally rehearse their own responses. This aligns with research by Johnson et al. [42], which, drawing on experiential, social, and cognitive learning theories, demonstrated that well-designed observer roles can be highly effective. According to the author, when combined with clear objectives, structured observation tools, and active participation in debriefing, observation can lead to learning outcomes comparable to those achieved through direct participation [42]. In the present study, undergraduate PT students had access to all these elements, which encouraged active engagement even when observing. Extending this perspective, a recent scoping review by Nojima et al. [43] highlighted that simulation experiences combining emotional engagement, structured reflection, and feedback not only foster skill acquisition but also enhance students’ intrinsic motivation and learning ownership—mechanisms equally reflected in the active observer role described by our participants. Consistent with this, students in our study expressed a strong desire for more frequent simulation experiences, suggesting that such engagement fosters sustained motivation and a sense of ownership in learning. Taken together, these findings indicate that learning in simulation emerges through multiple roles and perspectives (acting, observing, and reflecting), each contributing to the development of confidence, motivation, and professional identity.

## Strengths and limitations

This study presents several strengths, including its rich, theory-informed qualitative design that captured in-depth perspectives of PT students engaging in high-complexity SBE. Methodological rigour was further enhanced through a structured study design, piloting of the interview guide and by using member checking through transcript validation by participants, which strengthened the trustworthiness of the data. Additionally, the sample size was appropriate for qualitative research, and participant selection ensured both heterogeneity and homogeneity, providing diverse perspectives while maintaining coherence in the data.

The SBE examined in this study represents a distinctive strength: it is embedded as a longitudinal, compulsory component of the PT curriculum, aligned with the strategic educational framework of the degree. Therefore, simulations are implemented sequentially across multiple courses from the second to the fourth academic year, allowing for progressive complexity, continuity, and integration of learning.

Previous research has often focused on specific domains such as communication skills, empathy, decision-making, or confidence, often framed within self-efficacy or clinical preparedness. However, few studies have explored how students perceive their overall learning process in SBE. Recognising self-perceived learning as a unique, multifaceted outcome may provide deeper insight into how students internalise knowledge and integrate simulation into their professional development.

However, the study also has limitations. The single-institution setting may limit transferability of findings to other physiotherapy programs with different curricular structures, resources, or cultural contexts. The findings are based exclusively on student perspectives; future research could strengthen triangulation by incorporating additional viewpoints, such as those of facilitators, SPs (actors), and external observers, as well as objective performance measures.

Although FGs were conducted by researchers who were not teaching participants at the time of data collection, all interviewers were faculty members within the institution. This may have introduced some degree of social desirability bias or response inhibition despite efforts to create a neutral and non-evaluative environment.

In addition, participants were drawn from different academic years and therefore had varying cumulative exposure to simulation, which may have influenced the depth, confidence, or comparative framing of their reflection during the FGs. At the same time, this heterogeneity enriched the analysis by allowing exploration of perspectives across different stages of training and levels of prior simulation exposure.

Finally, although the analysis remained grounded in participants’ accounts, the use of experiential learning theory as an interpretive framework may have shaped analytic attention toward particular dimensions of learning, while alternative theoretical lenses may have foregrounded different aspects of the simulation experience.

## Recommendations for practice and research

- Educators should design SBE to include a gradual increase in complexity and incorporating both active and observer roles throughout academic courses.
- Facilitators training should focus on prebriefing strategies, non-judgmental communication, and scaffolding student learning through constructive feedback and debriefing.
- Future simulations could integrate dialogical feedback from SPs to provide students with insight into the patient’s emotional experience.
- Future research should continue to examine the impact of different simulation roles and formats across training stages to optimise the integration of SBE in PT education.
- Further research is needed to define, measure, and validate students’ self-perceived learning as a distinct construct across different educational contexts and learner roles.

## Conclusion

The present findings underscore the educational and professional relevance of embedding high-complexity SBE as a longitudinal and intentional component ofwithin undergraduate PT training. By situating SBE within Kolb’s experiential learning framework, this study demonstrates how structured opportunities for active participation, observation, and guided reflection foster not only technical and relational competencies but also the development of professional confidence and readiness for clinical practice.

To maximise its impact, SBE should be designed to include progressive complexity, repetition, and role alternation—allowing students to engage both as physiotherapists and as observers within psychologically safe learning environments. Equally important, facilitators must be adequately trained to guide learning processes, promote emotional safety, and sustain reflective dialogue.

## Supplementary Information

Supplementary Material 1: Appendix 1. Interview guide for the focus groups.

## Data Availability

The data supporting the findings of this study are available from the corresponding author upon reasonable request.

## Abbreviations

FGs: Focus groups
INACSL: International Association for Clinical Simulation and Learning
PIER: Planning, Implementation, Evaluation, Revision
PT: Physiotherapy
SBE: Simulation–Based Education
SP: Standardised Patient
SRQR: Standards for Reporting Qualitative Research
UVic: UCC–University of Vic–Central University of Catalonia
