# Supplementary material for: Physiotherapy students’ experiential learning through curriculum-integrated high-complexity simulation: a qualitative study
Source: Adv Simul (Lond). 2026 Aug 3;11:83. doi: 10.1186/s41077-026-00474-3 (PMC13729358; doi:10.1186/s41077-026-00474-3)
Supplement: Supplementary file 1 [file 41077_2026_474_MOESM1_ESM.docx]

**Appendix 1.** Interview guide for the focus groups.

*Notes for the interviewer*

- At the beginning, if you agree, we can give each participant a piece of paper to write down their name and place it in front of them, in case it helps us remember your names and address you more personally.
- A brief individual introduction: each participant says their name and a short description, for example, what year of the degree they are in or which case they were assigned during the simulation.

*Interview guide*

1. What role did you play during the most recent simulation? (observer or PT, individual for 3^rd^ and 4^th^ year students, or in pairs for 2^nd^ year students).
2. What did you learn from participating as a physiotherapist? And as an observer?
3. If you remember, could you explain how the simulation session unfolded? (including prebriefing and debriefing).
4. Do you recall any activities carried out during the prebriefing?
5. What was your experience during the debriefing?
6. Had you had any prior relationship with the facilitator (professor) leading the simulation? How do you think this previous relationship—or lack of it—influenced your experience?
7. How did you feel during the simulation session? What has been your personal experience with simulations?
8. What do you think was the purpose of the simulation within the course?
9. Could you describe any learning outcomes you gained from participating in the simulation?
10. How did the simulation help you connect what you learned in class (theory) with clinical reasoning or future professional practice?
11. Would you identify any advantages of including simulations in the PT curriculum? Which?
12. Do you think participating in the simulations influenced your confidence or preparedness for future clinical placements? In what way?
13. Would you highlight any elements of the simulation that could be improved?
14. How did the performance of the actor/actress influence your experience with the simulations?
15. Regarding the facilitator’s ability to guide the session, minimise criticism, etc., how did their facilitation influence your experience with the simulations?
16. Did you identify any situations or experiences at an emotional level during the simulation? (direct question about being emotionally overwhelmed). If yes: how did it unfold, and how did you experience the support provided by the facilitator?
17. What is your opinion about the spaces where the simulations took place? Did these aspects influence your experience in any way?
